# Wall-free droplet microfluidics for probing biological processes by high-brilliance X-ray scattering techniques

**DOI:** 10.3389/fmolb.2022.1049327

**Published:** 2022-11-16

**Authors:** G. Marinaro, R. Graceffa, C. Riekel

**Affiliations:** ^1^ Department of Biomedical Engineering, Lund University, Lund, Sweden; ^2^ Department of Mechanical Engineering, Graduate School of Engineering, The University of Tokyo, Bunkyo, Japan; ^3^ European XFEL, Schenefeld, Germany; ^4^ ESRF, The European Synchrotron, Grenoble, France

**Keywords:** droplet microfluidics, biological processes, synchrotron radiation, free electron laser, microbeam, nanobeam, X-ray scattering

## Abstract

Here we review probing biological processes initiated by the deposition of droplets on surfaces by micro- and nanobeam X-ray scattering techniques using synchrotron radiation and X-ray free-electron laser sources. We review probing droplet evaporation on superhydrophobic surfaces and reactions with substrates, basics of droplets deposition and flow simulations, droplet deposition techniques and practical experience at a synchrotron beamline. Selected applications with biological relevance will be reviewed and perspectives for the latest generation of high-brilliance X-ray sources discussed.

## Introduction

Manipulating of droplets in an open environment is as subset of digital microfluidics (DMF) (Abdelgawad and Wheeler 2009). One of DMF’s principle interest for probing biological processes by synchrotron radiation (SR) and X-ray free-electron laser (XFEL) scattering techniques is a reduction in sample consumption as compared to continuous flow microfluidics (CFM), used for example in “serial femtosecond X-ray crystallography” (SFX) based on “gas dynamic virtual nozzle” (GDVN) random crystallite delivery (Chapman et al., 2011; Spence 2020; Schriber et al., 2022). Indeed, while liquid volumes moved in CFM chips are in the μL to mL range, DMF volumes are rather in the nL to pL range. DMF-based sample environments favour therefore studying samples available only in small quantities, such as precious proteins, or probing structural processes in microscale volumes. Developing microscopic models for biological processes following droplet impact, evaporation and reactions is of interest for many biotechnological applications such as tissue engineering, interactions of protein molecules with ligands or virus particles with substrates. Probing molecular interactions during droplet coalescence is also important for areas such as cloud or emulsion formation where models are frequently based on high-speed digital camera images (Pirouz Kavehpour 2015).

As compared to droplets suspended in inert carrier liquids (Bringer et al., 2004; Opalski et al., 2019; Echelmeier et al., 2020), droplets evaporating in an open environment allow studying molecular transformations for a large concentration range or reactions with substrates. The absence of absorption and scattering from confining walls favors the observation of weak scattering features. Precise, drop-on-demand (DOD) deposition on surfaces corresponds to a printing process and inkjet technology used for SR or XFEL experiments (see below) is also used for mass printing applications (Hoath 2016).

**FIGURE 1:**
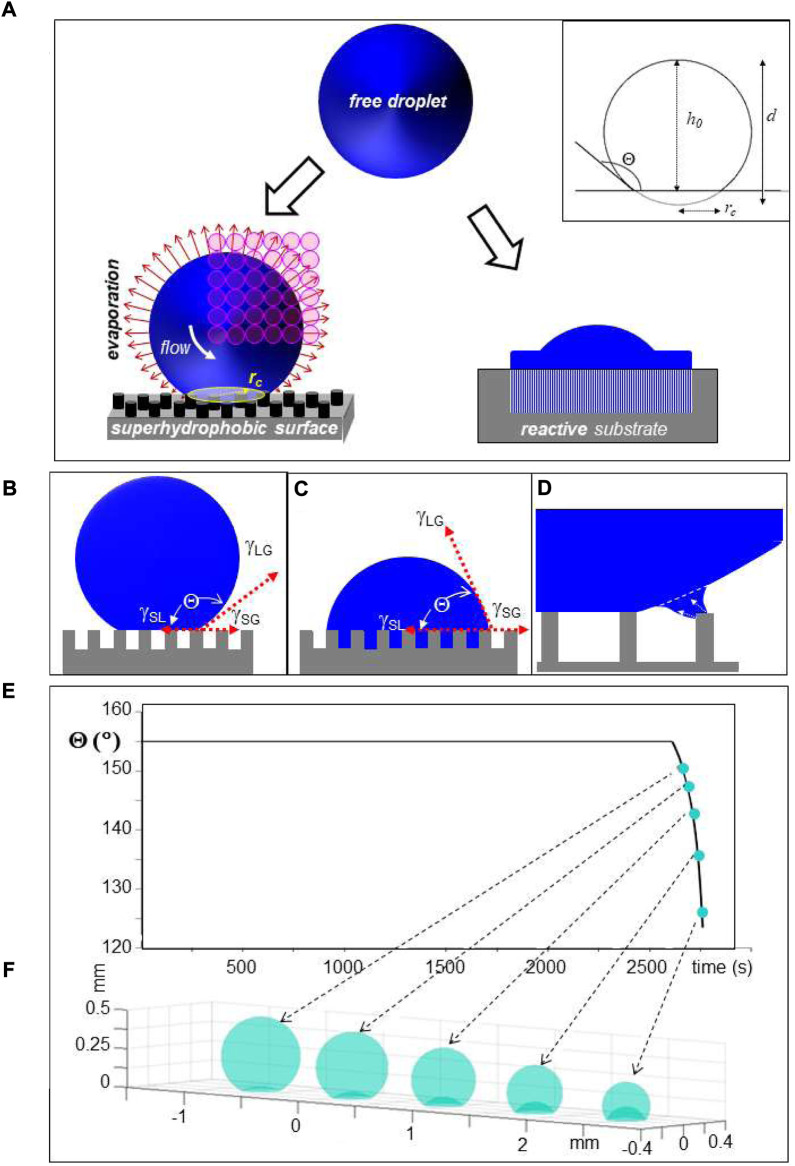
**(A)** Schematic design of droplet deposition. Left: Droplet deposited on a pillared SHS with a circular contact area of radius *r*_*c*_. The inset shows schematically the geometry of a deposited droplet. Selected geometric parameters are indicated in Table 1. Bulk flow is induced by surface evaporation. A mesh of SR probing positions is depicted by open circles depicting the focal spot. Right: Cut through droplet which has partially spread on surface with low Θ-value and reacted with underlying substrate. **(B)** Unpinned Cassie–Baxter (“Fakir”) state of water droplet on pillared SHS. γ_SG_, γ_SL_ and γ_LG_ are the interfacial surface energies between solid (S), liquid (L) and gas **(G)** phases and Θ is the contact angle in thermodynamic equilibrium (Accardo et al., 2014). **(C)** Pinned Wenzel state. **(D)** Retraction of contact-line of the droplet from the outermost pillars of a pillared SHS during evaporation (Allione et al., 2021). **(E)** Simulation of change in Θ for a 6 μL droplet during evaporation at r. t. The decrease of Θ above t∼2,600 s is due to the wetting transition. **(F)** Simulation of droplet shape change during wetting transition; E/F: adapted from: (Accardo et al., 2014).

Here we will review two prototype transformation routes following droplet deposition. Indeed, aqueous droplets deposited on a water-repellant, superhydrophobic surface (SHS) with a high contact-angle Θ [Figure 1A(left), inset] can be conceptionally considered as close-to wall-free confinements for water, aqueous solutions or suspensions including crystallites. Such droplets can serve as micro-reactors for probing molecular aggregation, assembly or transformations including chemical reactions by X-ray scattering and complementary techniques (Accardo et al., 2013a; Accardo et al., 2014; Allione et al., 2021). We will also review the deposition of droplets on surfaces with low Θ-values, with subsequent spreading and interaction with the bulk substrate by diffusion and/or reaction [Figure 1A(right)]. The review will highlight micro- and nanobeam X-ray scattering probes based on small-angle and wide-angle X-ray scattering (SAXS/WAXS; SWAXS when combined) techniques, revealing the emergence of hierarchical structural organization from nano-to meso- (>50 nm) scale. Experience with adapting inkjet droplet generation technology to a SR beamline will be discussed and ongoing technological developments of interest to SR/XFEL applications highlighted. Selected applications will be reviewed and perspectives for the latest generation of SR/XFEL sources discussed. For a review on probing ballistic droplets in flight see: (Graceffa and Accardo, 2018).

## Methods

**Droplets on SHSs.** Sessile droplets on SHSs retain a close to spherical shape (<1% shape distortion due to gravitational forces) outside a circular contact area of radius *r*_*c*_ but lose their rotational and translational degrees of freedom due to weak interactions with the surface roughness in the contact area (Accardo et al., 2014). *r*_*c*_ is defined by the sessile droplet’s volume *V*_*s*_ and Θ as: $r_{c}=\sqrt[{3}]{\frac{6V_{s}}{3\pi \tan\left(\frac{\Theta }{2}\right)+\pi {\left(\tan\left(\frac{\Theta }{2}\right)\right)}^{3}}}$ while the droplet’s height above the surface (*h*_*0*_) is: $h_{0}=r_{c}\tan\left(\frac{\Theta }{2}\right)$ (Hu and Larson 2002). Typical droplet parameters for a *d* = 30 μm diameter droplet (14 pL) from a piezo-actuated glass capillary inkjet-head (see below) are shown in Table 1.

**TABLE 1:** Parameters for a 14 pL droplet on a SHS with Θ = 150° contact angle. *V*_*s*_: volume, *F*_*f*_: surface area of free droplet, *F*_*c*_: contact area. *F*_*c*_ */F*_*f*_ is independent of the volume for the geometry shown in Figure 1A (inset); (parameters calculated by G.M.).

*d* (μm)	*V*_*s*_ (pL)	*r*_*c*_ (μm)	*F*_*c*_ (μm^2^)	*F*_*f*_ (μm^2^)	$\frac{F_{c}}{F_{f}}$ (%)	h_0_ (μm)
30	14	7.5	710	2,800	∼25	28

Droplets of several μL volume can be deposited by manual pipettes while smaller volumes down to the pL range and below require precise control of volume, position and timing of deposition available from DOD print-heads (Lee 2003; Hoath 2016). Indeed, ballistic droplets ejected at ≤10 kHz frequency from a piezo-driven inkjet propagate in air with speeds (*v*) up to several 10th m/s. While such droplets will not splash upon impact due to the dominance of surface tension (Lee 2003), the dynamics of impact on a SHS results in shape and contact line oscillations (Brown et al., 2011). Analysis of fluid dynamics during inkjet printing of microarrays for genotyping applications is relevant for droplet impact implying stronger droplet interactions with the substrate [Figure 1A(right) (Dijksman and Pierik 2008)].

The droplet’s shape is maintained during evaporation on a SHS in the “Cassie–Baxter” (also called “Fakir”) state (Figure 1B). The retraction of the three-phase contact-line induces, however, transient local deformations of the droplet’s shape at the surface roughness resulting in shear flow, which is particularly well observable for pillared SHSs (Figure 1D and see below). Shear flow induced at the droplet’s rim by evaporation (Figure 1A) differs from extrusion flow induced in the confined environment of the pillars (Figure 1D), which can be used for generating different nanofibrillar morphologies following biomolecular assembly (see below). The droplet shape adapts to the surface roughness at the wetting transition into the “Wenzel” state (Figure 1C) (Papadopoulos et al., 2013; Accardo et al., 2014). This transition can be simulated analytically based on a diffusion model as shown in Figures 1E,F(Popov 2005; Accardo et al., 2014). While the solution concentration can be increased by evaporation up to residue formation, CFM chips with a dialysis section provide only a limited increase in concentration (Junius et al., 2020).

Evaporation times of water droplets on a non-wetting surface depend strongly on the volume with ∼1,400 s for ∼5 μL (*d* ∼ 2,120 μm), 20 s for ∼1 nL (*d* ∼ 125 μm) and ∼5 ms for ∼1 fL (*d* ∼ 1.24 μm). Cooling times of ∼5 ms for droplets in the pL range (Ando et al., 2018) correspond to time-scales accessible to mass-spectrometry or stopped-flow techniques (Graceffa et al., 2013), allowing probing enzyme catalysis or protein aggregation. Cryo-SEM shows that cryo-frozen droplets on biological SHSs retain their Fakir state features (Ensikat et al., 2009). Whether faster protein dynamics such as conformational transitions could become trapped in cryo-frozen outer layers of droplets remains to be explored. Probing small volumes of laser beam molten/revitrificated cryo-frozen droplets (Voss et al., 2021) by scanning nanobeam X-ray scattering could also provide access to sub-ms protein dynamics.

**FIGURE 2:**
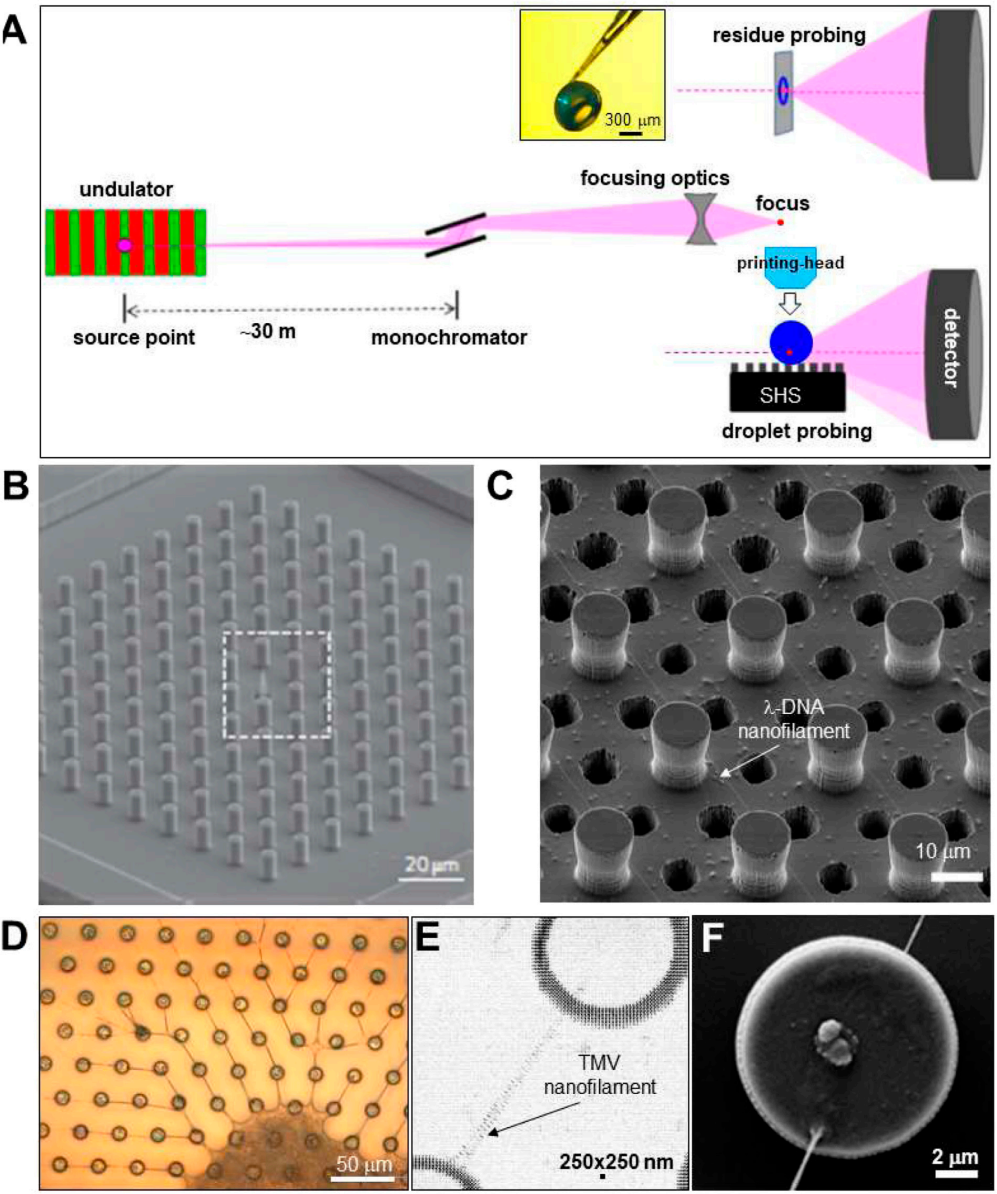
**(A)** Schematic design of focusing SR beamline. The pink undulator beam is monochromatized by a Si crystal and focused to the sample position. Two transmission scattering geometries are shown for (i) probing a droplet with the beam parallel to the SHS substrate and (ii) a sample with the beam normal to the substrate. Adapted from: (Marinaro et al., 2016). The inset shows a hollow, spherical lysozyme residue attached to a glass capillary tip (Accardo et al., 2011b). **(B)** SEM image of regular pattern of pillars with a central cone, etched into Si wafer. Adapted from: (De Angelis et al., 2011). **(C)** SEM image of pillared Si-SHS and 6 μm etched holes for imaging λ-DNA nanofilaments by TEM. Adapted from: (Gentile et al., 2012). **(D)** Optical microscopy image of TMV nanofilaments and central residue attached to SU-8 pillars on a Si_3_N_4_ membrane. **(E)** Density map obtained by mesh-scanning TMV nanofilament composed of two sub-filaments, spanning two SU-8 pillars. The contrast is provided by form factor scattering from the nanofilaments and interface refraction from the edge of the pillars. **(F)** SEM image of Si-pillar with attached nanofilaments. E/F: Adapted from: (Marinaro et al., 2014).

**Focusing SWAXS beamlines**. X-ray micro- and nanobeams can be generated by focusing the beam from a SR undulator source to the sample position by reflective, refractive or Fresnel lenses (Figure 2A) (Riekel et al., 2009). SR beamlines are usually providing monochromatic beams (ΔE/E∼2 × 10^−4^) although the use of a pink beam from an undulator harmonics (up to ΔE/E∼2 × 10^−2^ depending on harmonics) is possible. XFEL beamlines make generally use of the full band width of undulator harmonics (ΔE/E∼2 × 10^−3^). The brilliance of an undulator beamline allows keeping a reasonable divergence at the focal spot for SWAXS applications (Riekel et al., 2009). In practice, the SAXS resolution limit (Q_min_)^1^ corresponds to the beamstop cut-off, depending not only on source brilliance but also focusing optics and quality of the beam defining system. Indeed, the ESRF ID13 beamline provides after the EBS (extremely brilliant source) upgrade into a fourth generation SR source (Narayanan et al., 2022), a SAXS limit of Q_min_∼0.16 nm^−1^ (*d* ∼ 40 nm) for a ∼175 nm focal spot based on Si-refractive optics with a flux density of ∼1.3∗10^7^ photons/s/nm^2^ while sub-100 nm focal spots based on Fresnel optics with similar Q_min_ value are feasible (personal communication: M. Burghammer, ESRF-ID13). A complementary microbeam endstation with a ∼1.5 μm FWHM focal spot and ∼1.5∗10^5^ photons/s/nm^2^ flux-density based on refractive optics should allow further reducing Q_min_ due to a lower beam divergence (expected range: Q_min_∼0.06 nm^−1^; d_max_∼100 nm), well into the resolution range of third generation SR source SAXS beamlines. Scanning experiments are currently performed using fast framing pixel detectors in continuous-scanning mode, averaging across X-ray flashes emitted by the SR source (Graceffa and Accardo, 2018). Droplets deposited on a horizontal surface are probed in transmission geometry with the SR beam approximately parallel to the surface (Figure 2A). Residues on X-ray transparent substrates can also be probed in transmission geometry with the surface normal to the beam or tilted (Figure 2A). Larger residues can often be detached from a SHS and reattached to a glass-capillary for probing along arbitrary directions (inset Figure 2A).

XFEL SWAXS experiments with focused beams have been demonstrated for single amyloid fibrils at the Coherent X-ray Imaging (CXI) instrument of the Linac Coherent Light Source (LCLS) (Liang et al., 2015; Seuring et al., 2018). SAXS capabilities are also developed at European XFEL (EuXFEL) SPB/SFX beamline (Round and Mancuso 2022). This beamline provides a <300 nm focal spot (ΔE/E∼2 × 10^−3^) for a flux density per ∼25 fs pulse of >10^4^photons/nm^2^ or a ∼1.9 μm focal spot (Round and Mancuso 2022). While XFEL experiments are in general performed in vacuum environment, the SPB/SFX beamline provides an additional downstream interaction region with refocusing X-ray optics and helium environment which could be used for droplet applications. Scanning experiments with high lateral resolution across a sample are not possible as the sample volume probed by a single pulse is destroyed after the scattering process by radiation damage: “diffract and destroy” technique (Spence 2008). Instead, scanning techniques require probing successively fresh samples (e.g. mesh-scan of sample array). The high flux density enables in principle single droplet experiments (Graceffa and Accardo, 2018).

**Superhydrophobic surface (SHS) substrates** *.* Surface roughness mimicking biological SHSs, such as lotus leaves (Koch et al., 2009), can be fabricated in many ways from artificial materials. Indeed, plasma treatment of polymeric surfaces such as polymethyl methacrylate (PMMA) (Accardo et al., 2014) or polybutadiene (Brown et al., 2011) induces SHS properties. Polymeric substrates generate, however, often an unwanted diffuse X-ray scattering background. Microfabricated SHSs based on a microscale pattern of pillars etched into a Si wafer with a nonwetting surface layer (Figure 2B) generate a very low X-ray scattering background (Accardo et al., 2014; Gentile et al., 2014; Allione et al., 2021). A limitation of a planar array of homogeneous pillars is the lack of a stable position for a droplet in the Fakir state, requiring droplet pre-alignment in the focal spot and its tracking during evaporation. Droplet localization is, however, possible by pillar gradients (Shastry et al., 2006; Gentile et al., 2013) or by transforming a central pillar of a regular array into a cone (Figure 2B). The latter approach has allowed concentrating a 160 nL (*d* ∼ 310 μm) droplet of 1 fm lysozyme solution into a speckle of about five molecules at the tip of a cone (Gentile et al., 2010; De Angelis et al., 2011). Liquid crystalline phases forming at low solute concentration can also be used for extruding single DNA nanofilaments attached to the pillars from the retracting droplets interface (Figure 2C) (De Angelis et al., 2011). The low X-ray transmission of standard Si wafers does not allow probing a single nanofilament in normal-incidence transmission geometry (Figure 2A). Enhancing X-ray transmission by etching Si wafers results in fragile substrates. Atomic resolution images from DNA nanofilaments have, however, been obtained by transmission electron microscopy (TEM) through cylindrical or azimuthally extended holes or etched in a pillared Si-SHS (De Angelis et al., 2011) (Figure 2C). This approach can also be used for XRD with larger beams by averaging diffraction across several holes (Zhang and Moretti, 2020) although the restricted field-of-view does not allow for probing the attachment of a nanofilament to a pillar (Figure 2F). A higher substrate X-ray transmission can also be obtained by a microfabricated array of polymeric pillars on a Si_3_N_4_ membrane, as shown for nanofilaments extruded from tobacco mosaic virus (TMV) solution droplets (Marinaro et al., 2014; Marinaro et al., 2015) (Figures 2D–F). This type of SHS is, however, more challenging in fabrication and fragile in handling than Si-SHSs.

**FIGURE 3:**
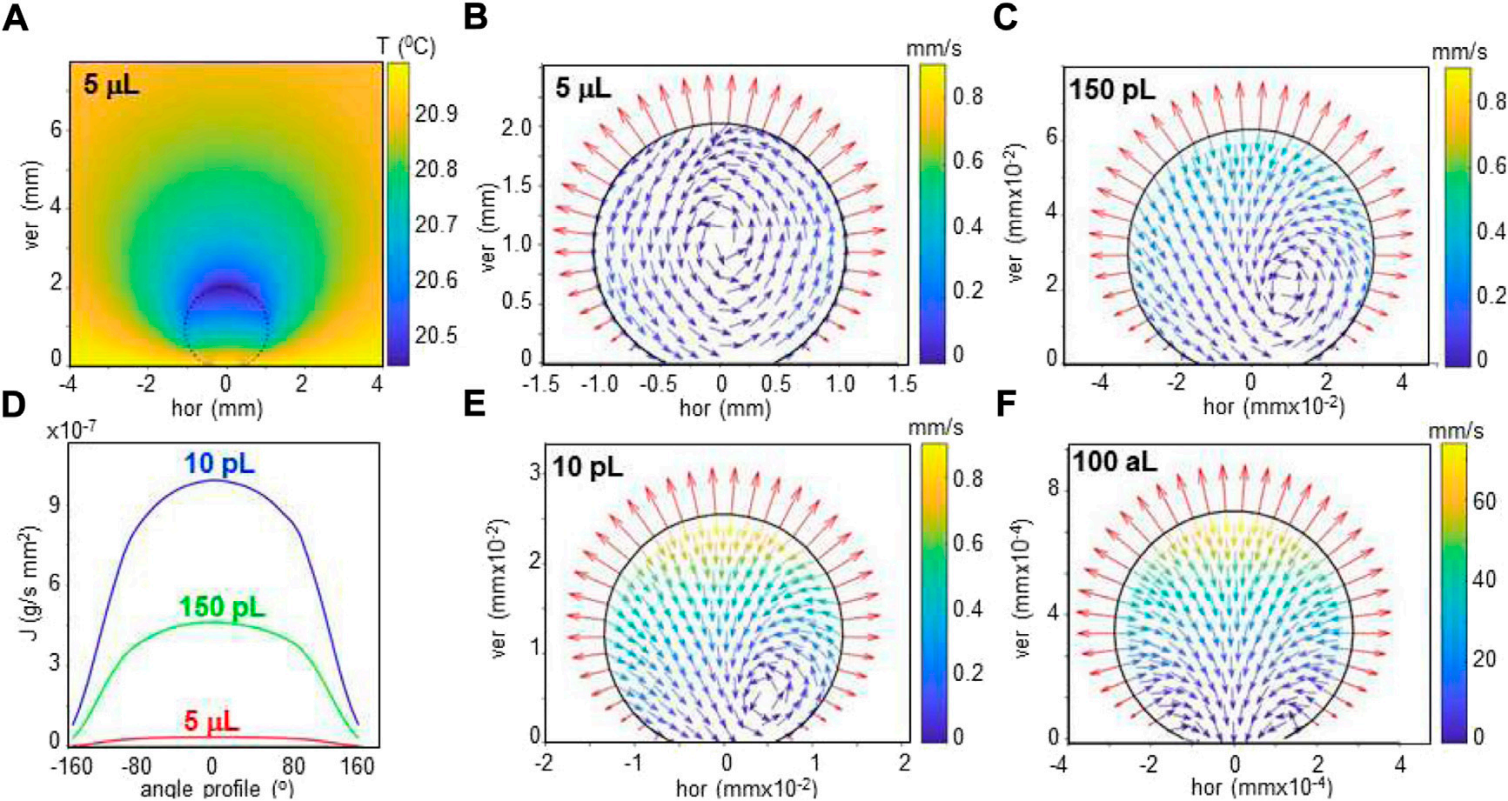
**(A)** Heat map of simulated temperature distribution of 5 μL droplet on SHS (dotted circle) and its environment at about 40% humidity. The temperature of substrate and distant surrounding air are set to 21°C. **(B)** Simulated evaporation flux along the rim of the droplet. **(C–F)**: Simulation of flow in aqueous droplet for 5 μL, 150 pL, 10 pL and 100 aL volumes. A recirculating flow is revealed by flow vectors for the 5 μL droplet. For smaller droplets, the radial flow increases and the recirculating flow center is pushed to the contact area. The evaporation flux vectors are normal to the rim. A/B/C adapted from: (Marinaro et al., 2021); D/E: **(G)** M., unpublished.

**Flow simulations** of sessile water droplets on SHSs based on Langevin’s equation and finite element methods (FEM) suggest that surface evaporation induces an anisotropic temperature gradient across the interface resulting in an evaporation flux along the rim which is strongly dependent on the droplets volume (Marinaro et al., 2021) (Figures 3A,B). The temperature gradient is the driving force for flow-fields generated in the droplets, such as the recirculating flow in a 5 μL droplet (Figure 3C). Flow-fields can be visualized in water droplets loaded with polystyrene particles by particle image velocimetry (PIV) (Marinaro et al., 2021). Simulated flow-vectors scale with droplet volume down to the aL range while the flow-field changes gradually from recirculating to radial below about 10 nL, accompanied by a shift of the center of circulation (Figures 3C–F). Radial flow without a center of circulation is modeled for 100 aL (*d* ∼ 0.29 μm) droplets (Figure 3F). The temperature gradient at the droplets interface could in principle be modulated by the humidity of the air surrounding the droplet (Accardo et al., 2014). As a side-line we mention that the flow field in a droplet can result in fractionation of a particles size distribution (Marinaro et al., 2021) with possible applications in serial crystallography (SX, SFX) experiments (see below).

**Droplet-on-demand (DOD) deposition** is frequently based on inkjet technology developed for mass printing applications (Lee 2003; Hoath 2016). We mention below, however, also other ejection techniques which have either been used for SR/XFEL applications or are promising approaches.

**FIGURE 4:**
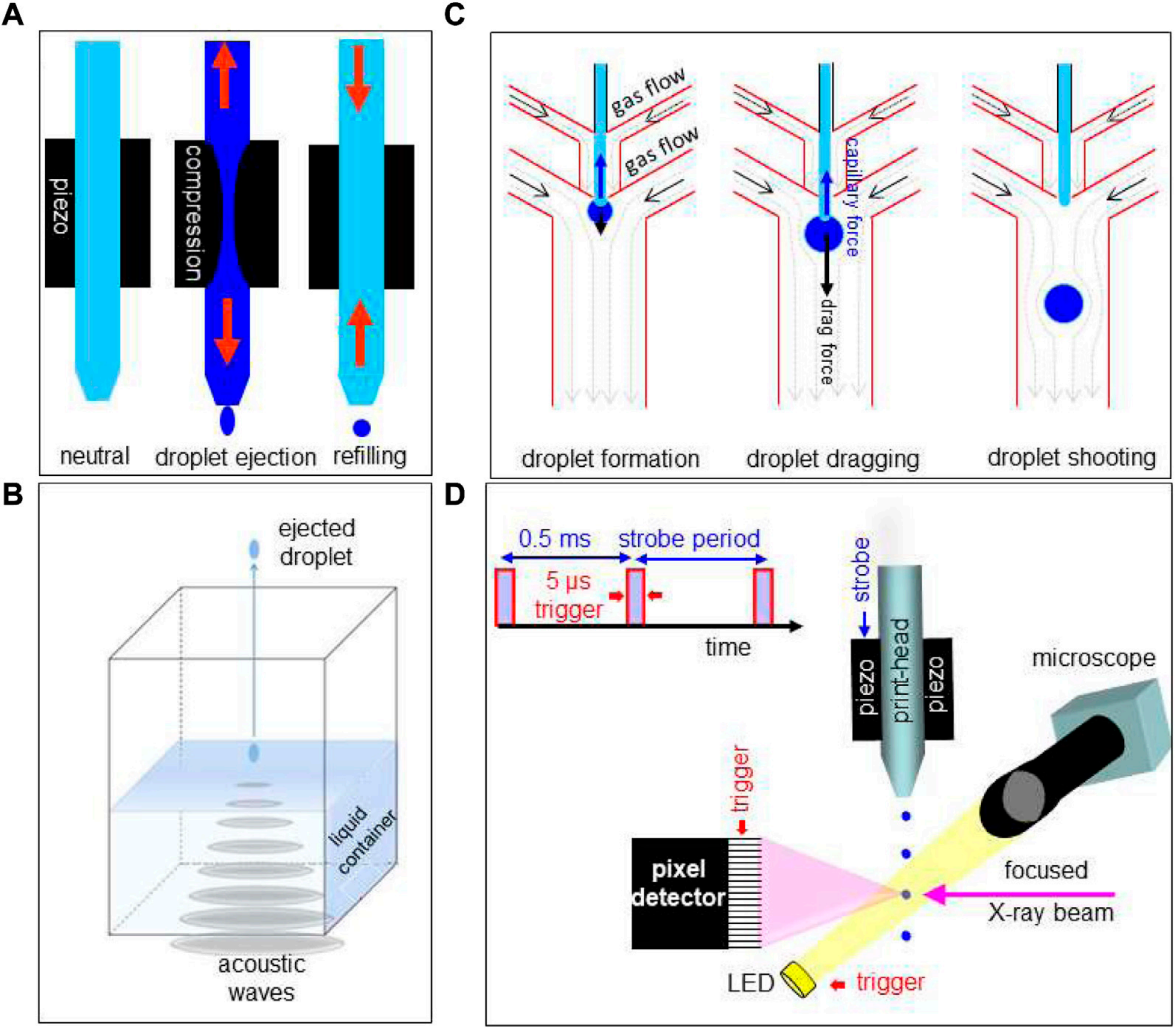
**(A)** Schematic operation sequence of DOD inkjet system. Adapted from: (Graceffa 2010). **(B)** Schematic design of acoustic droplet ejection (ADE) system. Adapted from: (Teplitsky et al., 2015). **(C)** Operating sequence for hybrid gas/liquid droplet generating system. Adapted from: (Kazoe et al., 2021). **(D)** Schematic design of DOD inkjet system integrated into a SR beamline. Strobe period and trigger of detector framing time are generated by electronic modules, integrated into the beamline control system. Droplets are ejected at 2 KHz by strobe-pulses acting on the piezo. During the flight of a ballistic droplet through the focused SR beam, the pixel detector frontend and the LED are activated by trigger-pulses. Adapted from: (Graceffa 2010).

*Inkjet systems* allow ejecting pL droplets with a defined frequency of up to several 10th of kHz from an inkjet-head nozzle by piezo-actuator compression (Figure 4A) or *via* internal fluid heating. Droplet ejection at room temperature is limited approximately to liquids with 10–100 times the viscosity of water (∼1 mPa^.^s) (Lee 2003; Hoath 2016). Commercial high-throughput printing systems are evolving towards manufacturing technologies derived from semiconductor industry such as MEMS-based inkjet-heads (Martin et al., 2016). For applications on temperature sensitive and potentially corrosive fluids, piezo-actuated glass capillary inkjets-heads or microfluidic DMF chips are preferable (Lee 2003; Hoath 2016). Indeed, piezo-actuated inkjet-heads allow ejecting droplets loaded with fragile biological objects such as single cells without degradation (The and Yamaguchi, 2013). Droplets with ultrahigh viscosities can be ejected from inkjet-heads by acoustic forces pulling at the nascent droplet. The so-called “acoustophoretic printing” technique (Foresti et al., 2018) has allowed printing ∼1 nL droplets with viscosities up to ∼25 × 10^3^ mPa^.^s, i.e. above the range of cytoplasmic viscosities of human eukaryotic cells (Wang et al., 2019).

Commercial DOD systems with a single glass capillary inkjet-head (Figure 4A) generate monodisperse droplets down to ∼14 pL (*d* ∼ 30 μm) (e.g. microdrop Technologies)^2^. At smaller diameters, aerodynamic effects and Brownian motion will increasingly perturb the trajectory (Lee 2003). Ultra-small droplet volumes in the aL range require a guiding potential between inkjet-head and substrate (electrohydrodynamic (EHD) jet) (Lee 2003). Indeed, ballistic droplet volumes of 14–65 aL (*d* = 0.064–0.108 μm) (Mishra et al., 2010) and 4.8 aL (*d* ∼ 0.045 μm) (Park et al., 2007) were extrapolated from the area of the imprints. 3D printing set-ups using EHD manipulation (pyro-EHD) have been applied to polymeric and biopolymeric materials (Coppola et al., 2015; Serdar Onses et al., 2015; Coppola et al., 2020).

*Acoustic droplet ejection (ADE)* allows ejecting droplets from the surface of a liquid in an open container. By focusing sound waves to the air-liquid interface, droplets are ejected into the air (Figure 4B). Problems with nozzles such as clogging are avoided (Roessler et al., 2013). Droplet ejection speeds and frequencies are comparable with piezo inkjet technology (Roessler et al., 2016).

*DMF droplet ejection* (called also “droplet shooting”) relies on microscale-flow in a hybrid gas/liquid DMF chip (Figure 4C) (Kazoe et al., 2021). The ejection process is based on a combination of capillary and drag forces acting on the droplet with reported ejected volumes of 3.61–24.9 pL at kHz frequencies (Kazoe et al., 2021). A modification of this approach is the introduction of a piezo-acoustic actuator based on kHz acoustic waves to control the Rayleigh instability for droplet generation (Laurell et al., 1999). We also note in this context that GDVN free liquid jets can be transformed into droplet streams by acoustic modulation of the coaxially co-flowing gas (DePonte et al., 2008). DOD operation is, however, not possible for such a system.

**Adapting a DOD set-up to a SWAXS beamline**. Modular DOD set-up components, based on an commercial piezo-actuated glass-capillary inkjet-head and control electronics allowing stroboscopic data collection, have been integrated into a SR beamline environment (Figure 4D) (Graceffa et al., 2008; Graceffa et al., 2009; Graceffa 2010; Graceffa et al., 2012; Mehrabi and Schulz, 2019). A motorized ink-jet head allows aligning ballistic or printed droplets in the X-ray focal spot and a motorized support platform is used for transferring the inkjet-system from a loading/servicing to a probing position (Graceffa 2010). Optical visualization normal to the X-ray beam direction allows verifying droplet trajectory and droplet impact position during data collection. Ballistic droplets in flight have to be visualized in stroboscopic mode while printed droplets remain visible during evaporation. Shielding of the droplet trajectory by a thin polymer foil is required to avoid perturbations by air. This approach allows in principle also controlling the humidity around the droplet by flow-through of humidified gas. (e.g.: ESRF-ID13)^3^. More practical details are reported in: (Graceffa 2010).

One of the main problems encountered in using such a system at a beamline with different scientific applications are ejection instabilities due to clogging of the nozzle, in particular for smaller droplets. This could be limited by an automated self-cleaning procedure, available for commercial DOD printing systems (Su et al., 2021). Replacing the inkjet-head nozzle by a superhydrophobic sieve has been proposed for avoiding clogging, in particular from suspensions (Modak et al., 2020). Alternative options are acoustic forces acting on the nascent droplet or EHD guiding fields. Integrated DOD systems with multiple print-head cartridges, controlled environment and self-cleaning procedures for various liquids are commercially available (e.g. Fuji film DMP-2850)^4^. Adaptation of a stand-alone system to a multiple user beamline environment is, however, challenging and has not yet been reported.

## Applications

Many experiments reviewed in this section were performed prior to the introduction of pixel detectors and dedicated nanofocusing X-ray optics for biophysical studies, limiting time- and lateral scanning resolution achievable (see: Perspectives).

**FIGURE 5:**
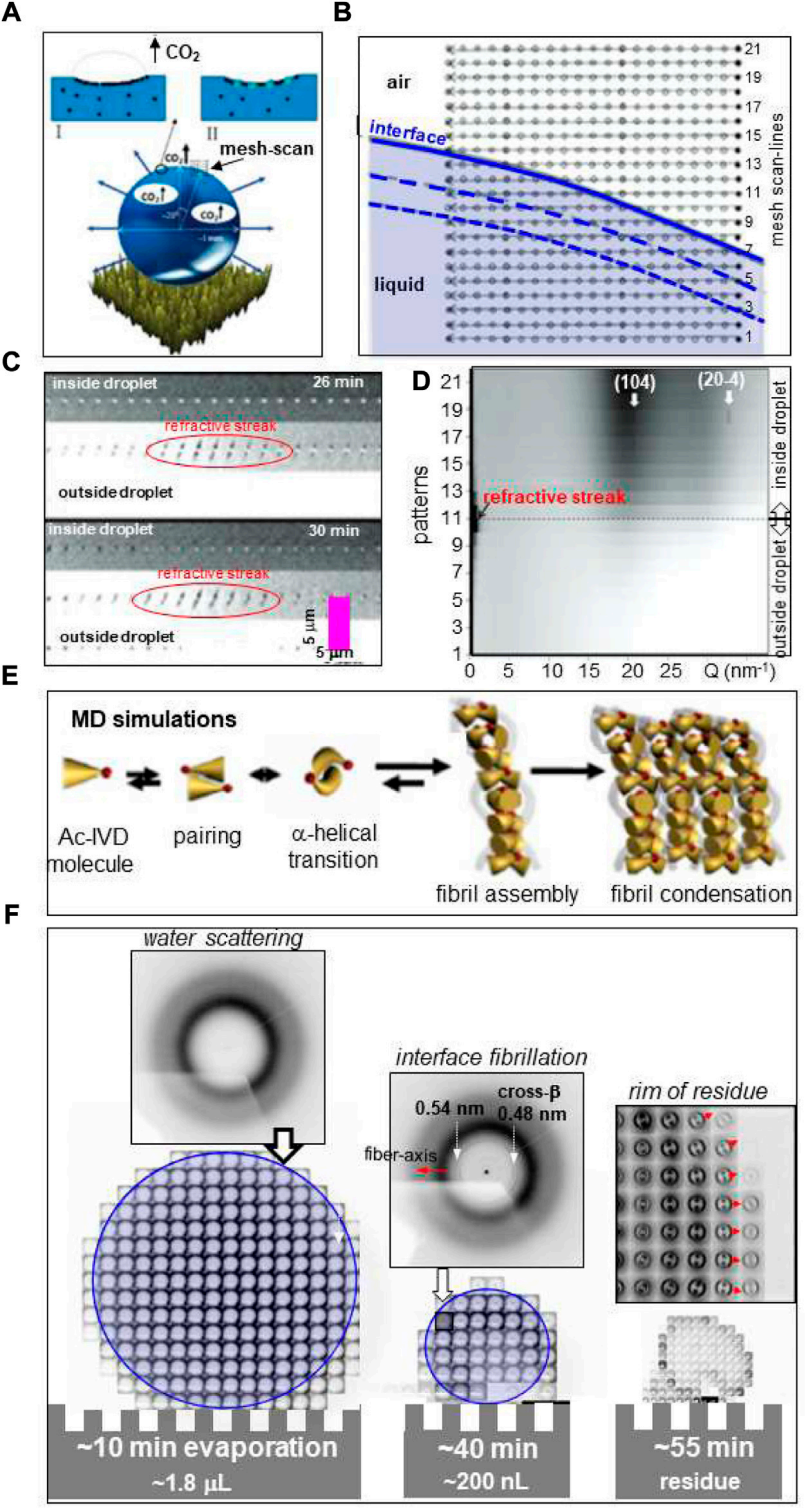
**(A)** Schematic picture of evaporating Ca(HCO_3_)_2_ solution droplet on PMMA surface showing surface accumulation of CaCO_3_ and evaporating CO_2_ bubbles. **(B)** Mesh-scan with 21 linear scan-lines of retracting droplet liquid/air interface through 1.7 μm focal spot with 0.5 s/frame. The interface at the start of evaporation is schematically depicted as solid blue line. The dashed blue lines indicate changes of the interface at t = 20/40 s. **(C)** Three scan-lines through interface area at two evaporation times with 0.1 s/frame. The refractive streaks indicate the position of the liquid/air interface. **(D)** Co-existence of diffuse scattering from amorphous CaCO_3_ and reflections of calcite at 26 min after start of evaporation. **(E)** MD simulation of Ac-IVD self-assembly. **(F)** Mesh-scan through ∼1 μm focal spot at three time points of evaporating 4 μL Ac-IVD solution droplet deposited by a pipette on a Si-SHS solution with 0.5 s/pattern (Marinaro et al., 2016). Amyloid fibrils are revealed at the rim after ∼40 min evaporation by a 0.48 nm cross-β peak. The 0.54 nm peak was tentatively attributed to an α-helical phase. Red arrows correspond to fiber axis direction. A/B/C/D: Adapted from: (Accardo et al., 2011b); **(E)** adapted from: (Hauser et al., 2011); **(F)** adapted from: (Marinaro et al., 2016).

**Droplet evaporation on SHS.** *Amorphous CaC O*_*3*_ (*ACC*) is the least stable modification of CaCO_3_ with important biological functions such as fast buildup of coral skeleton composed of crystalline CaCO_3_. Probing ACC formation and its conversion in confined environments is therefore of considerable interest. Exploring the development of hierarchical organization requires both WAXS and SAXS information. Indeed, scanning microXRD of a ∼4 μL Ca(HCO_3_)_2_ solution droplet on a PMMA-SHS, decomposing into CaCO_3_, H_2_O and CO_2_ (Figure 5A), reveals the formation of a dense ACC layer at the droplet’s interface preceding crystallization (Accardo et al., 2011a). The position of the retracting interface during evaporation was determined by refractive streaks allowing adapting the mesh-scans to the droplet volume (Figures 5B–D).

Spherical ACC particles were revealed by their form factor scattering during coalescence of CaCl_2_ and Na_2_CO_3_ solution droplets on an electrowetting-on-dielectrics (EWOD) device in open planar geometry with SHS coating and 10 Hz framing rate (Accardo et al., 2013b). The onset of ACC particles growth was observed at 400–500 ms after the onset of coalescence. No information on preceding precipitation steps, such as emulsion-type CaCO_3_ formation deduced by TEM from flash-frozen precipitates (Rieger et al., 2007) was obtained.

**FIGURE 6:**
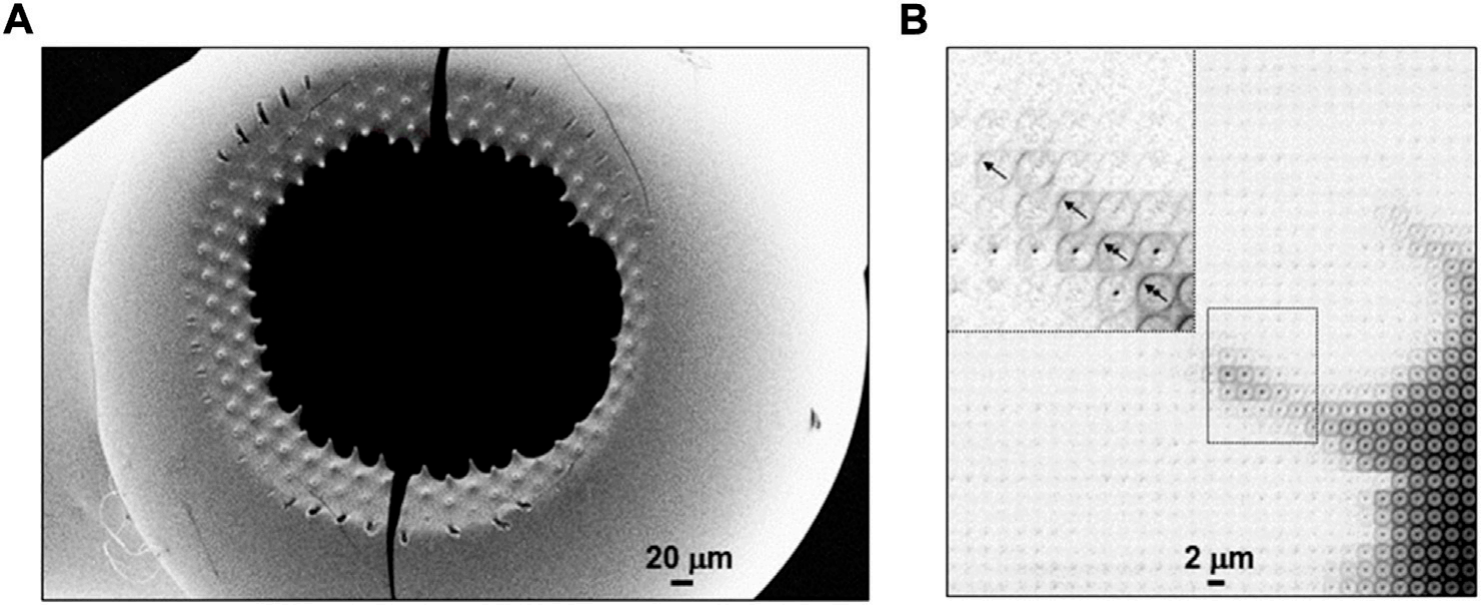
**(A)** SEM image of hollow lysozyme solution residue detached from pillared SHS substrate revealing pinning points. Adapted from: (Accardo et al., 2010; Accardo et al., 2011b). **(B)** Density map based on mesh-scan of two pinning points from β-amyloid (1–42) solution residue through ∼1 μm focal spot. Adapted from: (Marinaro et al., 2016). The inset shows the cross-β patterns in the outer part of one of the pinning points with arrows indicating the local fiber axes orientation. The outermost patterns show enhanced orientational ordering and crystallinity.

*Short oligo-peptides* have been shown forming fibrillar phases by increasing the concentration of aqueous solutions (Hauser et al., 2011; Lakshmanan et al., 2013). Molecular dynamics (MD) simulation suggests that fibrillation is preceded by α/β transition of peptide dimers (Figure 5E). Fibrillation of Ac-IVD peptide with the formation of an amyloid cross-β structure was observed *in-situ* by scanning nanoXRD for an evaporating droplet (Hauser et al., 2011). Mesh-scans across the evaporating droplet were limited to few time-points due to limitations in detector speed (Figure 5F) (Marinaro et al., 2016). The onset of fibrillation was observed at the rim of the droplet (Figure 5F). A mesh-scan of the detached residue reveals that the amyloid cross-β axis is aligned normal the circumference of the rim (Figure 5F) suggesting fibrillar self-assembly in a recirculating laminar flow-field of μL volume droplets (Figure 3C). The morphology of the residue reflects the formation of a dense fibrillar layer at the surface with pinning points contacting the SHS. Indeed, a SEM image of a detached hollow lysozyme residue reveals elongated pinning points (Figure 6A) (Accardo et al., 2010) suggesting frozen-in elongational shear flow at the transition into the Wenzel state (Figure 1D). This has a significant impact on crystallinity and orientational ordering as shown for a mesh-scan of two pinning points from a β-amyloid (1–42) solution residue (Marinaro et al., 2016) (Figure 6B). Enhanced fibrillation attributed to a convective flow field at the rim of droplet residues on SHSs as compared to hydrophilic surfaces was observed for a number of amyloid systems (Accardo et al., 2015). Convective flow ordering of 40–100 nm exosome particles into a lamellar morphology was also observed at the rim of droplet residues (Accardo et al., 2013a).

**FIGURE 7:**
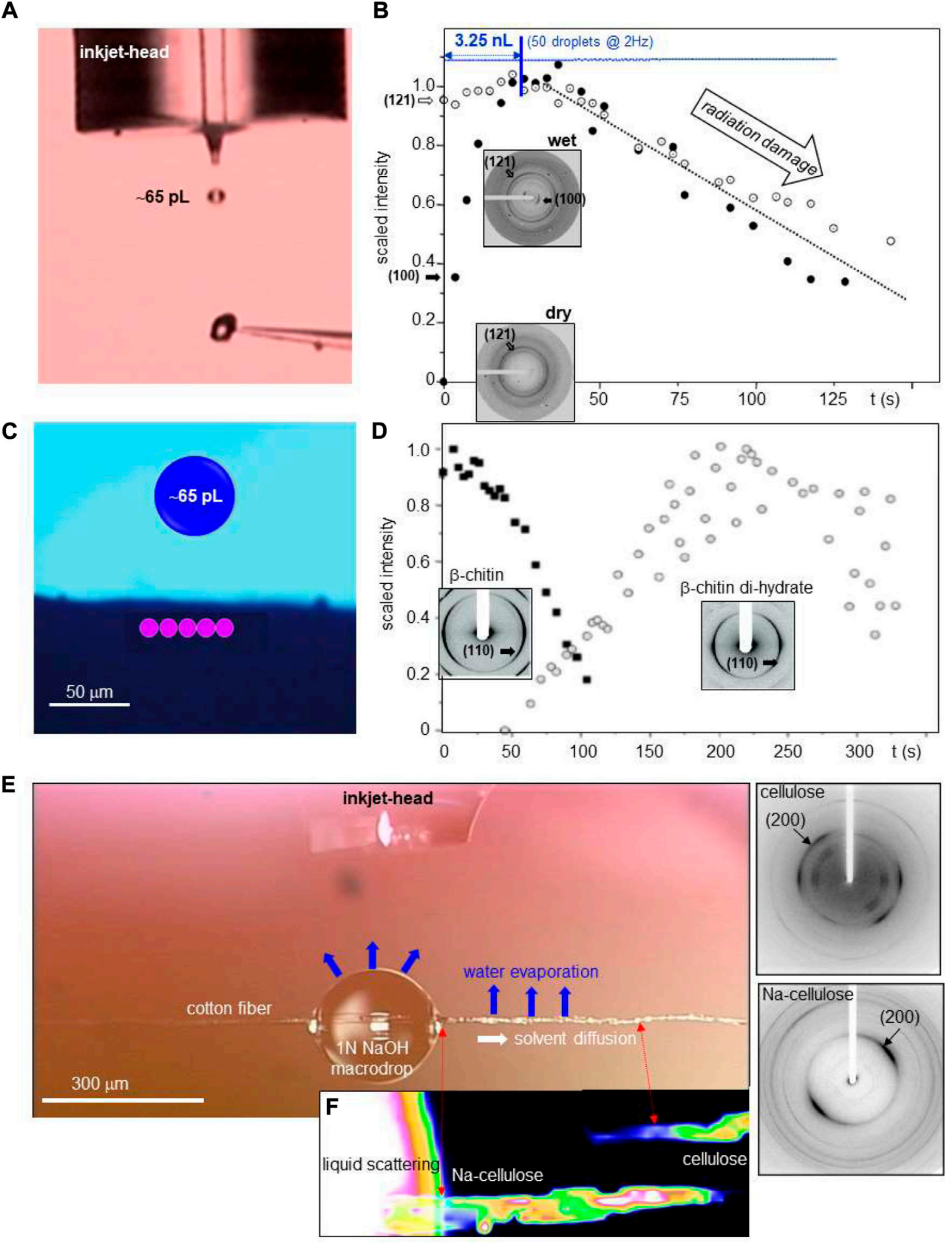
**(A)** Stroboscopic image of a ballistic water droplet directed towards a ∼30 μm diameter potato starch granule attached to a glass tip. **(B)** Structural kinetics of potato starch granule hydration based on 5 μm focal spot. The variation of (100)/(121) Bragg peak intensities is shown during printing of ∼65 pL water droplets at 2 Hz. Insets: patterns of dry and hydrated starch. The sequence of water droplets is shown schematically as a line of blue circles. The maximum of crystalline hydrate is reached for overall ∼3.25 nL water deposited. A/B: adapted from: (Lemke et al., 2004). **(C)** Schematic design of focal spot positions (in pink) on the sample and the size of a 520 pL droplet (in blue). **(D)** Structural kinetics of β-chitin hydration based on 10 μm focal spot. The variation of β-chitin and β-chitin di-hydrate (110) Bragg peak intensities is shown. C/D: adapted from: (Roessle et al., 2003). **(E)** Inkjet head above cotton fiber with deposited NaOH solution macrodrop. **(F)** Structural kinetics of Na-cellulose formation based on 300 nm focal spot. The heat map is based on (200) Bragg peak intensities of cellulose and Na-cellulose. The intensities are correlated with the volume concentration of both phases. Corresponding nanoXRD patterns are shown to the right. Adapted from: (Schoeck et al., 2007).

**Droplet reactions with substrate** *. Starch granules* are the most important energy reserve in higher plants. The structural change involved in hydration and gelatinization was probed for a ∼30 μm diameter starch granule, centered in a 5 μm focal spot (Lemke et al., 2004) (Figure 7A). ∼65 pL (*d* ∼ 50 μm) water droplets were printed at 2 Hz onto the granule and a sequence of 0.5 s microXRD patterns was recorded. The uptake of water by the lattice is correlated with the intensity increase of the (100) reflection while the (121) lattice reflection shows only weak dependence on hydration (Figure 7B) The hydrated phase reaches its maximum for ∼50 printed droplets, corresponding to ∼3.25 nL). Printing more droplets decreased the crystalline fraction due to secondary radiation damage by radicals generated by photoelectrons.

*β-chitin hydration* was probed for tubes providing the housing for *Birsteinia* deep sea worm (Roessle et al., 2003). The transformation of dry β-chitin into β-chitin di-hydrate was observed by printing ∼65 pL (*d* ∼ 50 μm) droplets at 10 Hz from an inkjet head (Figure 7C). The sample was displaced laterally in a repetitive way through the 10 μm focal spot by five steps in order to reduce local radiation damage. This allowed observing the transformation of β-chitin into its di-hydrate phase while the also known mono-hydrate phase could be excluded as intermediary phase (Figure 7D).

*Cellulose into Na-cellulose transformation* by alkaline solution is the first step of the commercial “mercerization” process. This transformation was probed by printing 65 pL (*d* ∼ 50 μm) droplets of 1N NaOH solution into a 300–400 μm diameter “macrodrop” on a cotton fiber (Figure 7E) (Schoeck et al., 2007). The solution diffuses along the fiber becoming increasingly concentrated along its path by H_2_O evaporation, transforming cellulose into Na-cellulose above the NaOH concentration limit. A heat map based on the (200) reflections of cellulose and Na-cellulose reveals, however, an increasing Na-cellulose concentration from the macrodrop up to the phase limit with unreacted cellulose (Figure 7F). This can be understood by an initial accumulation of a nonstoichiometric Na-cellulose phase at the phase limit. The further NaOH concentration increase in the evaporating macrodrop shifts the Na-cellulose concentration gradient towards the macrodrop (Schoeck et al., 2007).

**Droplets for serial X-ray crystallography (SX)** allow reducing sample consumption and background scattering while distributing radiation damage on multiple small protein crystals. SR time-resolved SR (TR-SX) has been demonstrated for probing ligand binding to microcrystallites, such as hen egg-white (HEW) lysozyme and xylose isomerase, at the EMBL P14 beamline (DESY-Hamburg) (Mehrabi and Schulz, 2019). A piezo-driven DOD inkjet system deposited ∼75 pL droplets of ligand solution on a multiwell crystallization chip with 2 × 10^4^ crystallites of ∼20 μm diameter each. As compared to CFM chips, sample consumption is drastically reduced to <0.2 μM/chip. The droplet volume is about nine times the volume of a single crystallite, reducing significantly background scattering. Translation of the chip through the beam was synchronized with data collection of 10 ms exposure to reduce radiation damage. Full ligand occupancy was observed in 0.1–1 s.

**FIGURE 8:**
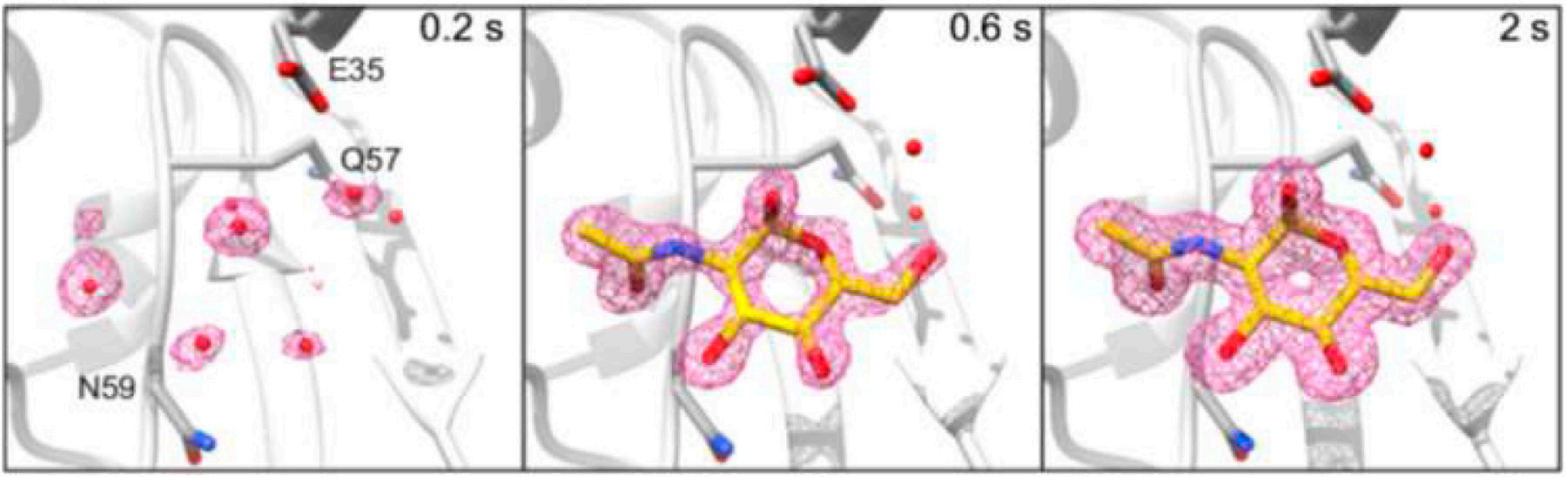
*2mF*_o_–*DF*_c_ electron density maps for HEWL-GlcNAc Structures, displayed at ±1*σ* contour level and carved at 0.2 nm around the GlcNAc ligand. The GlcNAc molecule from the 2 s time point structure was used to carve the 200 ms time point map. All maps are at 1.45 Å resolution. Adapted with permission from: (Butryn et al., 2021).

A complementary approach consists in using an ADE print-head to deposit droplets containing protein microcrystallites on a conveyor belt based on hydrophobic polyimide bringing them into the X-ray probing region. Exploratory XFEL (TR-SFX) experiments were performed at the LCLS XPP beamline for 5–400 μm diameter crystallites of lysozyme, thermolysine and other proteins (Roessler et al., 2016). Microcrystallites, suspended in 0.1–2.5 nL droplets, were synchronized with the X-ray flash arrival. Adding a reaction area between ADE print-head and X-ray probing region allowed probing photochemical reactions and chemically triggered reactions by reactive gas of metalloenzymes such as photosystem II using combined SFX and X-ray emission spectroscopy (XED) at the LCLS MFX beamline (Fuller et al., 2017). XFEL TR-SFX experiments on reactions of HEWL lysozyme and serine β-lactamase with ligands such as N-acetyl-D-glucoamine for HEWL were performed by combining ADE and inkjet print-heads at BL2 beamline of SACLA(Butryn et al., 2021). About 3 nL-size crystal-containing droplets, deposited by an ADE print-head on the conveyor belt, were exposed to bursts of 150 pL droplets of ligand solutions by a piezo inkjet-head in drop-on-drop mixing mode. The mixture was further transported to the X-ray probing region allowing obtaining time-resolved structures with time points from 200 ms to 2 s (Figure 8) with an estimated lower limit of 50 ms. Overall sample consumption was as for the SR-SX experiment (Mehrabi and Schulz 2019) very low with 0.7 μM protein and 0.18 ml (41 μM) ligand for the HEWL sample.

## Perspectives

*SR applications*: Adapting specific droplet deposition experiments to multiple user beamlines requires disposable print-heads with integrated reservoirs and cleaning systems. At least two DOD print-heads have to be synchronized allowing depositing droplets with different solutes for co-printing applications. Print-heads could be based on inkjet technology and fabricated by two-photon 3D printing techniques, analogue to CFM chips (Calvey et al., 2019) or DMF chips for crystal-loaded droplets in inert liquids developed for SX/SFX (Echelmeier et al., 2019; Echelmeier et al., 2020). Given the temporal and spatial precision of droplet deposition, a support structure for sequential probing of evaporating droplets could be based on a rotating cartridge with small deposition areas and auto-centring SHS features (e.g., Figure 2B), providing a large opening angle for scattered photons in transmission geometry. Dedicated print-heads for specific applications could require a different technology. Indeed, acoustophoretically printed (Foresti et al., 2018) viscous droplets could serve as injection media for fixed target SX/SFX (Kovácsová et al., 2017). An array of crystal-loaded viscous droplets could be printed in a calibrated sample loading position and mesh-scanned with the supporting X-ray transparent membrane oriented normal to the beam. This approach could also be used for ultrafast mapping of combinatorial libraries of biomaterials generated by inkjet printing (Li et al., 2020).

Scanning SWAXS could be performed for smaller droplet volumes, higher lateral scan-resolution and shorter scan-times than experiments performed prior to the upcoming of pixel detectors and SR source brilliance upgrade. Indeed, a 20 × 20 points interface scan of CaHCO_3_ solution droplets (Figure 5B) of ∼200 s exposure time could be reduced to <1 s, allowing tracing convective flow fields *via* scattering from particles or aggregates (Accardo et al., 2013b). The increased data collection rate requires on-line software tools for on-line droplet centering (e.g. *via* interface streaks) (Graceffa et al., 2009), continuously adapting scan-ranges to changes in droplet size and tracking of nascent scattering features like Bragg peaks. For probing reactive processes in droplets, radiation damage has to be distributed across multiple droplets at different time-points after the onset of evaporation.

The concept of droplets as close-to wall-free micro-reactors (Accardo et al., 2014; Allione et al., 2021) allows exploring and optimizing specific reaction pathways, supported by flow simulations and complementary spectroscopic as well as imaging techniques. The power of this approach has been demonstrated for amyloid fibrillation of Tau441 protein molecules. A temperature-gradient driven, Marangoni-type flow in μL droplets provided a quasi-confined local environment, resulting in well-ordered fibrillar pockets at the droplet interface (Zhang and Moretti, 2020).

*XFEL application*: DOD print-heads developed for SR applications could also be used for XFEL applications, in particular for probing ultrasmall droplet volumes or contact line dynamics during droplet coalescence (Graceffa and Accardo, 2018). These authors discuss also options to mitigate droplet explosions producing fragments perturbing neighbouring droplets shape and trajectory in a train of droplets (Stan et al., 2016). An important subject could be probing nucleation processes. Megahertz hard X-ray microscopy based on the contrast provided by spatial coherence and flux of XFEL pulses (Vagovic et al., 2019) could reveal fast stochastic density fluctuations at the onset of density fluctuations preceding nucleation. Limiting self-assembly of polymeric and biopolymeric molecules to few nucleation events and growing chains provides exciting perspectives for probing reaction pathways with unprecedented precision. The challenge will be developing dedicated droplet printing set-ups allowing *in-situ* probing of the formation of nanofilaments (Figures 2C–F) at a retracting contact-line (Figure 1D) in a repetitive way and synchronized with XFEL pulses.

## Footnotes

1 Q = 4πsinΘλ^−1^ = 2π*d* ^−1^ where Θ: Bragg angle, λ: wavelength, *d*: lattice spacing.

2
www.microdrop.de/microdrop-dispenser-heads.html.

3
www.esrf.fr/UsersAndScience/Experiments/XNP/ID13.

4
www.fujifilm.com/us/en/business/inkjet-solutions/inkjet-technology-integration/dmp-2850.
